# Huge Renal Angiomyolipoma Mimicking a Renal Cell Carcinoma: A Case Report

**DOI:** 10.7759/cureus.30036

**Published:** 2022-10-07

**Authors:** Poosapati D Devasilpa Raju, Rajesh G Gattani, Govind Nagdev

**Affiliations:** 1 Surgery, Datta Meghe Institute of Medical Sciences, Wardha, IND; 2 General Surgery, Jawaharlal Nehru Medical College, Wardha, IND; 3 Emergency Medicine, Datta Meghe Institute of Medical Sciences, Wardha, IND

**Keywords:** ultrasonography, histopathological analysis, computed tomography, benign smooth muscle tumour, renal angiomyolipoma

## Abstract

Renal angiomyolipoma is a benign mesenchymal tumor composing adipose tissue, smooth muscle, and blood vessels. Despite being benign, the tumor sometimes can be aggressive with a locoregional and venous extension. Here, we present a case of a 48-year-old female who presented with a lump in the abdomen for two months, which was initially small and gradually increased in size associated with pain. Ultrasound of abdomen-pelvis showed ill-defined heterogeneous lesion arising from left kidney showing few hypoechoic and calcific focus within it. Contrast-enhanced computed tomography suggested an exophytic mass lesion with a central scar and calcification measuring 13 x 11 cm indicating renal cell carcinoma. Based on the benign nature and being well encapsulated, the patient was taken up for a left radical nephrectomy. However, the final histologic assessment concurred with renal angiomyolipoma.

## Introduction

Renal angiomyolipoma (AML) is the most common benign mesenchymal tumor composing adipose tissue, smooth muscle, and blood vessels [1]. The prevalence of AML varies between 0.2% and 0.6% with female preponderance [2]. Nearly 80% of these tumors are sporadic [2]. However, in a few cases, this is associated with tuberous sclerosis complex [2]. Despite being benign, the tumor sometimes can be aggressive with a locoregional and venous extension [2]. Due to abundant adipose tissue, they show a characteristic appearance on imaging and therefore can be easily identified [3]. However, sometimes they lack detectable adipose tissue, which makes it difficult in distinguishing them from renal cell carcinoma (RCC) [2]. Management of AML is based on clinical presentation and should be customized for every patient ranging from active observation to more invasive approaches [3]. Therefore, an accurate preoperative diagnosis of renal AMLs is crucial to prevent unnecessary nephrectomies and preserve renal functions [4]. Here, in this study, we report a similar renal AML case mimicking renal cell carcinoma in a 48-year-old female who underwent radical nephrectomy.

## Case presentation

A 48-year-old female presented with complaints of a lump in the abdomen for two months which was initially small and was gradually increasing in size. She described the abdominal lump as associated with pain around the lump and it was diffuse and dull aching in nature. She further described the pain as non-radiating with no aggravating or relieving factors. The patient never had any other complaints of fever, urinary tract infections, hematuria, or dysuria in the past.

**Figure 1:**
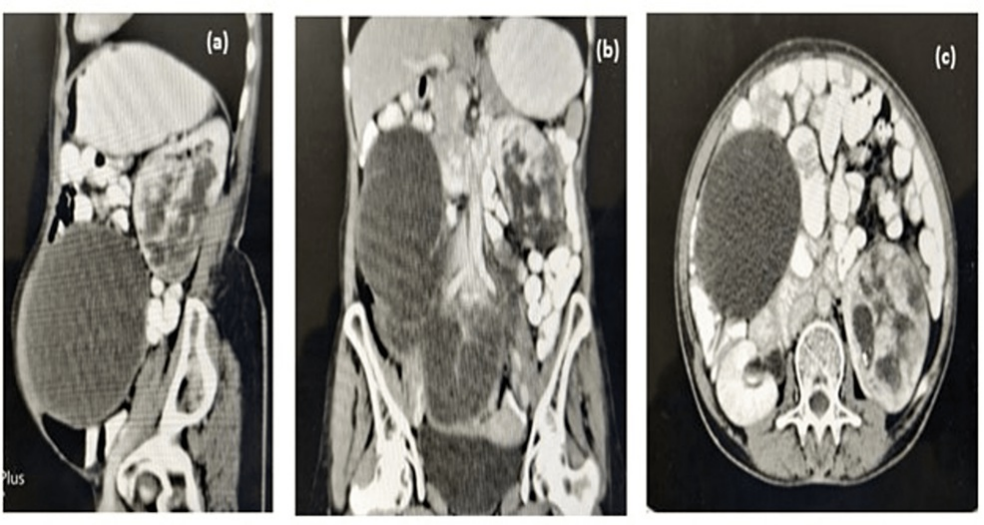
CECT images (a, sagittal plane; b, coronal plane; c, axial plane) showing 13 x 11 cm mass of the left kidney. CECT: contrast-enhanced computed tomography

Physical examination revealed an abdominal lump of size 13 x 11 cm seen in the left hypogastric region, which was extending to the umbilical region, with firm, non-tender, diffuse margins, smooth surface with no local rise of temperature, or no skin changes over the abdomen. Ultrasound abdomen-pelvis showed features suggestive of an ill-defined lesion arising from the left kidney's upper pole, showing few hypoechoic and calcific focus within it. Contrast-enhanced computed tomography of the abdomen and pelvis suggested a well exophytic hyperechoic mass lesion measuring 13 x 11 cm with central scar and calcification, indicating the possibility of renal cell carcinoma with no lymphadenopathy or adjacent organ invasion depicted in Figures 1a-1c.

**Figure 2:**
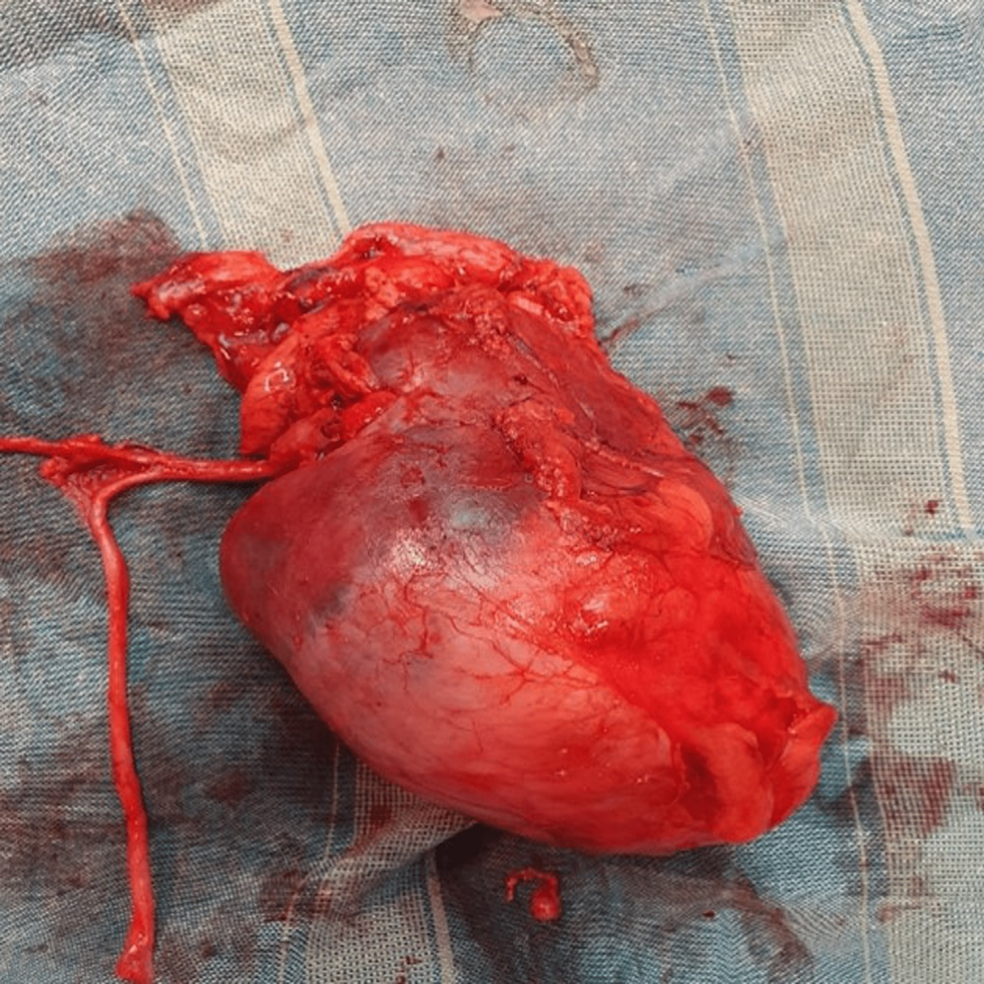
Gross findings of the resected tumor.

USG-guided biopsy of RCC was done for which histopathology reports showed that they were inconclusive. Histopathological analysis of the section from the kidney peritumoral area revealed chronic pyelonephritis. A section of the tumor showed features suggestive of angiomyolipoma. A section from Gerota fascia showed fibrous blood vessels and a congested vessels section from the capsule shows unremarkable fibrous tissue. A section from perinephric fats showed unremarkable adipose tissue with a congested blood vessel section from Gerota fascia showing fibrous blood vessels and congested vessels. A section from the capsule showed unremarkable fibrous tissue. Malignant epithelial negative for infiltration. A section from perinephric fats showed unremarkable adipose tissue with the congested blood vessel. Hence, based on the clinical and radiological findings, diagnosis of a possible benign RCC was made and the patient was taken for a left radical nephrectomy. The procedure was uneventful and the tumor was well encapsulated with no adhesions with the adjacent organs. The image of the left radical nephrectomy specimen is depicted in Figure 2.

**Figure 3:**
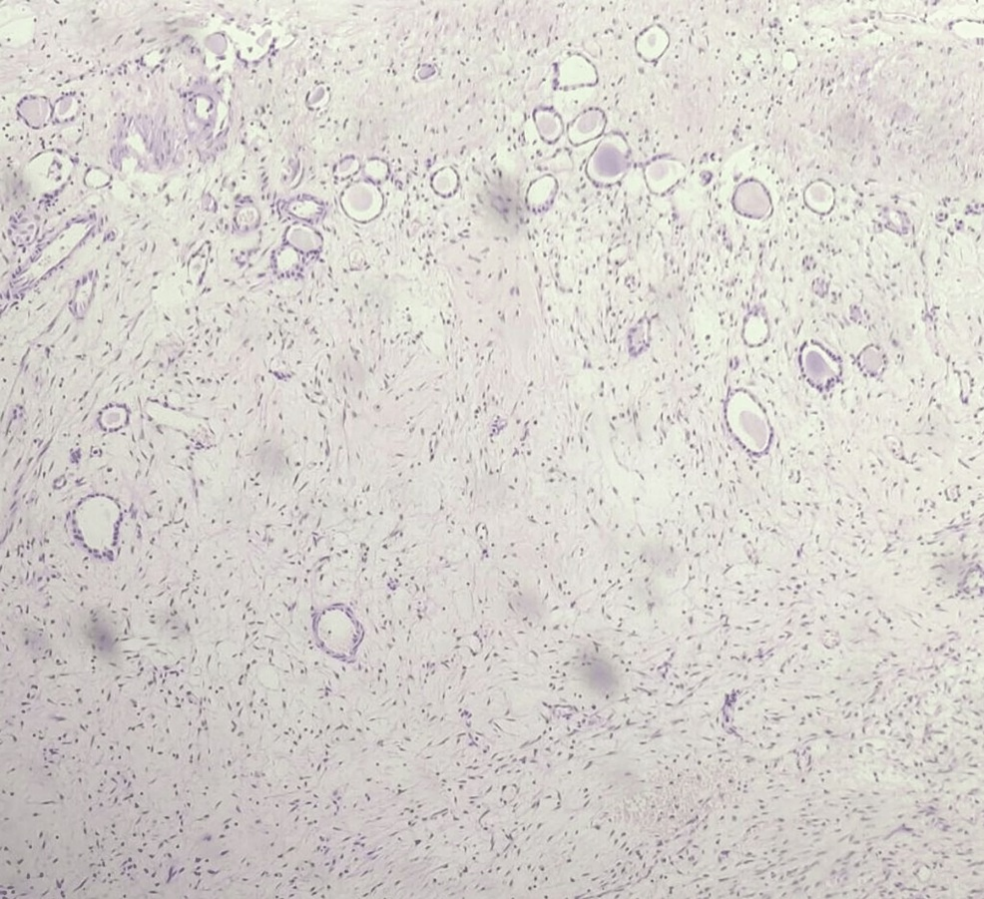
Histopathology slide shows multiple abortive tubules at the periphery with colloid-like material and myoid components.

However, the postoperative period was uneventful. At the time of discharge, the patient was hemodynamically stable with a healthy surgical site with no gape or distention. Histopathology of the resected specimen was suggestive of a renal angiomyolipoma as depicted in the image (Figure 3).

## Discussion

Literature reported that classic renal AML can be differentiated from RCC by the presence of macroscopic fat in sufficient quantity by contrast-enhanced computed tomography (CECT). Nevertheless, in a few of the AML cases fat cannot be visualized on CT making it difficult to differentiate from RCC [4]. Hence, sufficient clinical and imaging information is required to diagnose fat-poor and fat-invisible AML. In this study, we reported such a renal AML case along with clinical, radiologic, and histopathological findings and treatment.

Consistent with our case, few other studies reported the prevalence of renal AML to be higher in middle-aged women [1-5]. Clinical information supporting a diagnosis of AML includes a younger age, female sex, and asymptomatic presentation [3]. However, in our study, the middle-aged female patient presented with a firm, non-tender abdominal lump in the left hypogastric region extending to the umbilical region.

Renal AMLs were typically hyperechogenic on baseline ultrasonography [6]. While other studies reported that only 88% of RAMLs and 32% of RCCs also showed hyperechogenicity on ultrasonography [7]. Therefore, hyperechogenicity could not be used for the definitive diagnosis of renal AML. With respect to imaging findings, the absence of calcification, multiple lesions, perinephric collateral vessels, and hyper attenuation in comparison to renal parenchyma on CECT might raise the suspicion of AML [3]. The CECT features of heterogeneous enhancement, and an enhanced peritumoral rim highly suggest RCC, whereas homogeneous enhancement and prolonged enhancement manifest renal AML [7]. However, in our study, the ultrasound of the abdomen-pelvis showed an ill-defined heterogeneous lesion arising from the upper pole of the left kidney showing few hypoechoic and calcific focus within it. While CECT findings show a well exophytic mass lesion with central scar and calcification suggesting RCC. Hence, histopathological analysis was conducted for a definite diagnosis.

In a study conducted by Al Umairi et al., the histological features of minimal fat, necrosis, and hemorrhage suggested renal AML [8]. In our study, histopathological features of chronic pyelonephritis, fibrous blood vessels of Gerota fascia, and congested vessels of capsule confirmed renal AML. Similar to our case, other studies also confirmed renal AML by histopathological analysis without any immunohistochemistry [1-9].

Embolization, nephron-sparing surgery, complete nephrectomy, cryo- and radio-frequency ablation, and treatment with mammalian target of rapamycin (mTOR) inhibitors are the treatments available for renal AML [3]. However, surgical excision of early-stage AML confined to the kidney through partial or radical nephrectomy is the treatment of choice to remove the renal mass and prevent the recurrence from other parts of the body [3]. Furthermore, nephrectomy is suggested when a renal AML is very large and when suspicion of malignancy is high. Hence, in our study, the patient with renal AML confined to a kidney with a large tumor size underwent a radical nephrectomy to remove the benign renal tumor. Hajjaj et al. study also stated a 36-year-old male patient with a large renal mass >35 cm underwent radical nephrectomy in a transabdominal approach [9].

## Conclusions

The most common benign tumor is renal AML but due to its heterogeneous nature confirmatory imaging, histopathological analysis, and surgical excision are to be performed when an AML is suspected in the differential diagnosis to reduce significant morbidity and mortality.
